# Supplementary material for: CXCL12 and MYC control energy metabolism to support adaptive responses after kidney injury
Source: Nat Commun. 2018 Sep 10;9:3660. doi: 10.1038/s41467-018-06094-4 (PMC6131511; doi:10.1038/s41467-018-06094-4)
Supplement: Supplementary file 1 — Supplementary Information [file 41467_2018_6094_MOESM1_ESM.pdf]

Supplementary Information

Supplementary Figures

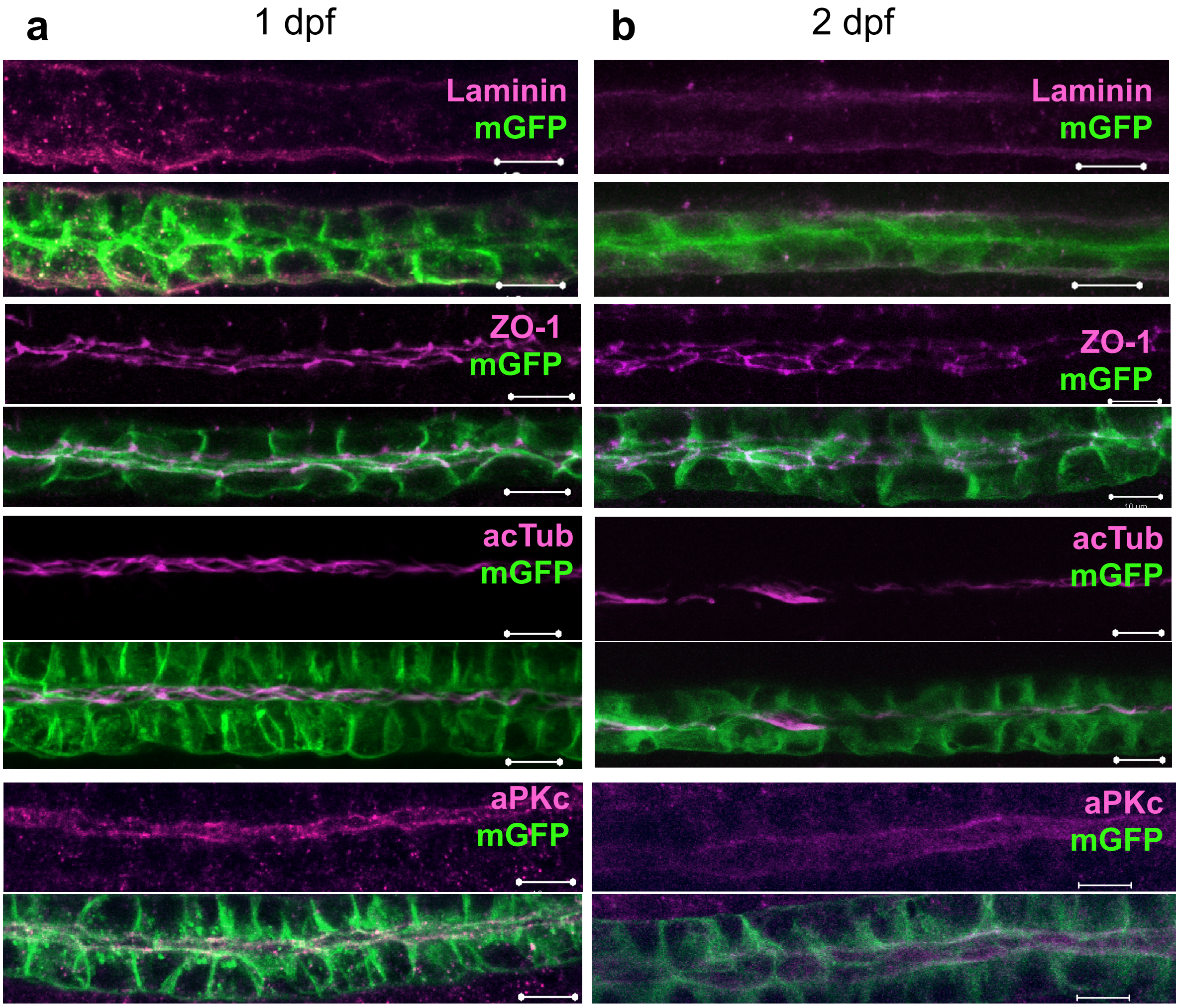

Supplementary Figure 1 | No apparent differences between the zebrafish pronephros at one and two days post fertilization. The pronephric tubules were morphologically similar in one-day- **(a)** and two-day- **(b)** old zebrafish embryos. Antibody staining for components of the extracellular matrix (laminin), tight junction complex (ZO-1), cilia (acetylated tubulin), and aPKC did not reveal morphological differences (Scale bars, 10 μm).

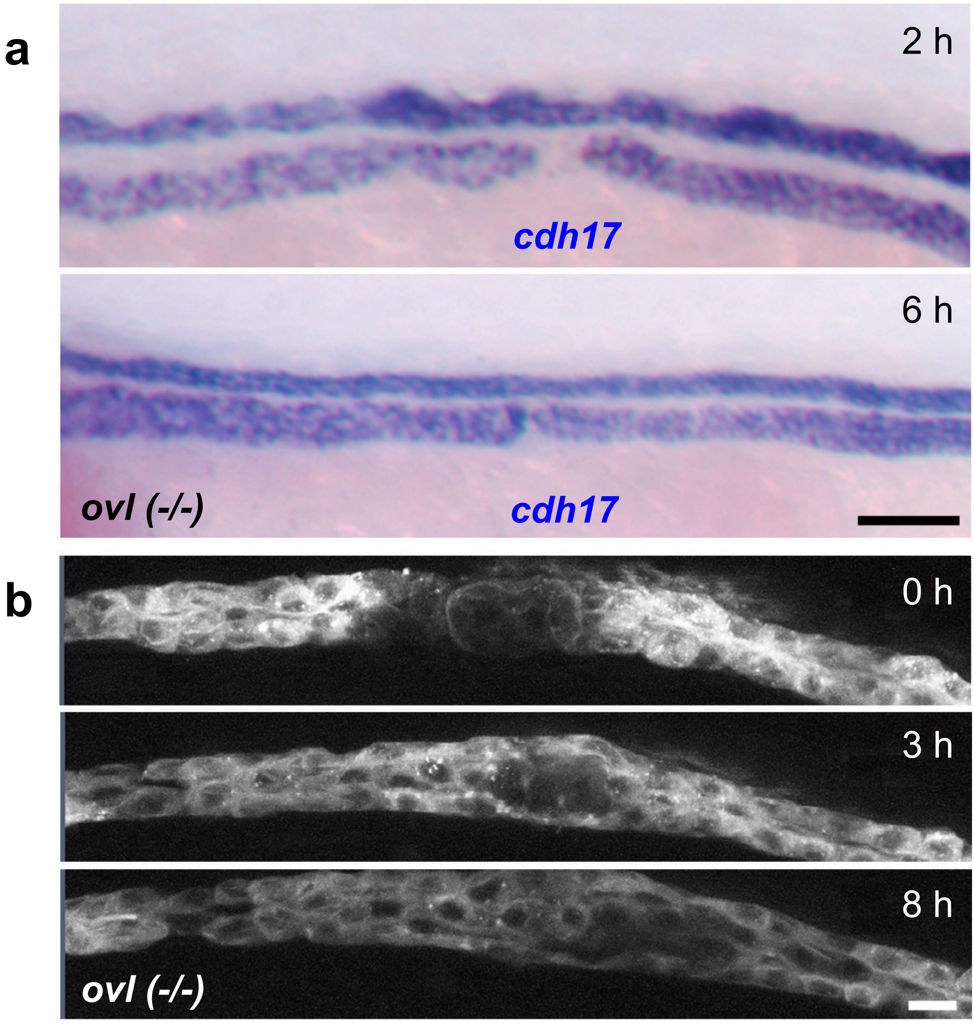

Supplementary Figure 2 | Cilia and fluid flow are not required for pronephros repair

(a) Homozygous *ift88(-/-)* (*ovl*) zebrafish embryos were injured 3 days after fertilization, and fixed 2 and 6 hours after wounding. *In situ* hybridization was performed with the pronephros-specific marker *cadherin17* (*cdh17*). At this stage, the embryos displayed prominent cysts and duct dilatations due to ciliary defects. The gap, clearly visible at 2 hours, was repaired 6 hours after injury. The results were confirmed in three independent experiments with at least 3 control and 3 mutant embryos (Scale bar, 100 µm). **(b)** Frames from a time-lapse movie following the recovery in *ift88(-/-)* (*ovl*) embryo. The laser-induced injury was completely repaired over an 8-hour time course (Scale bar, 10 µm).

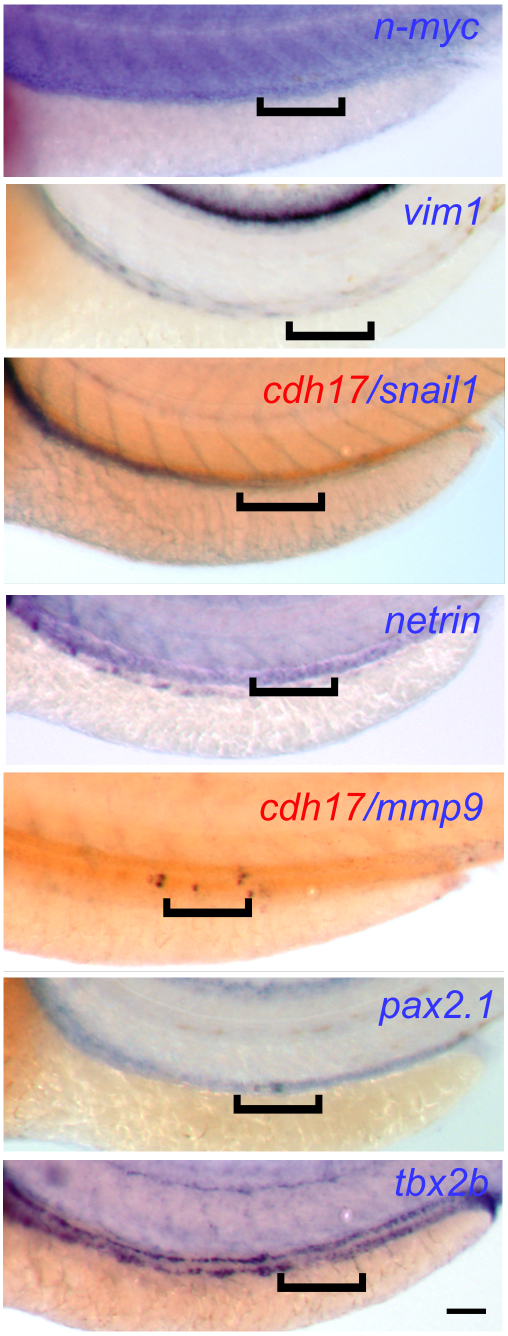

Supplementary Figure 3 | Pronephros repair is independent of EMT.
In situ hybridization for genes characteristic for epithelial-to-mesenchymal transition (EMT) or de-differentiation. Two-day-old embryos were injured, fixed 4 hours after wounding, and stained for EMT-typical genes (*N-myc, vimentin, snail, netrin* and *mmp9*), or for genes characteristic for non-differentiated pronephric cells (*pax2.1* and *tbx2b*). None of the markers showed up-regulation in the repair region, outlined by a black bracket. The staining for *mmp9* and *snail* was performed in combination with staining for *cdh17* labeling the pronephric cells in red (Scale bar, 100 µm).

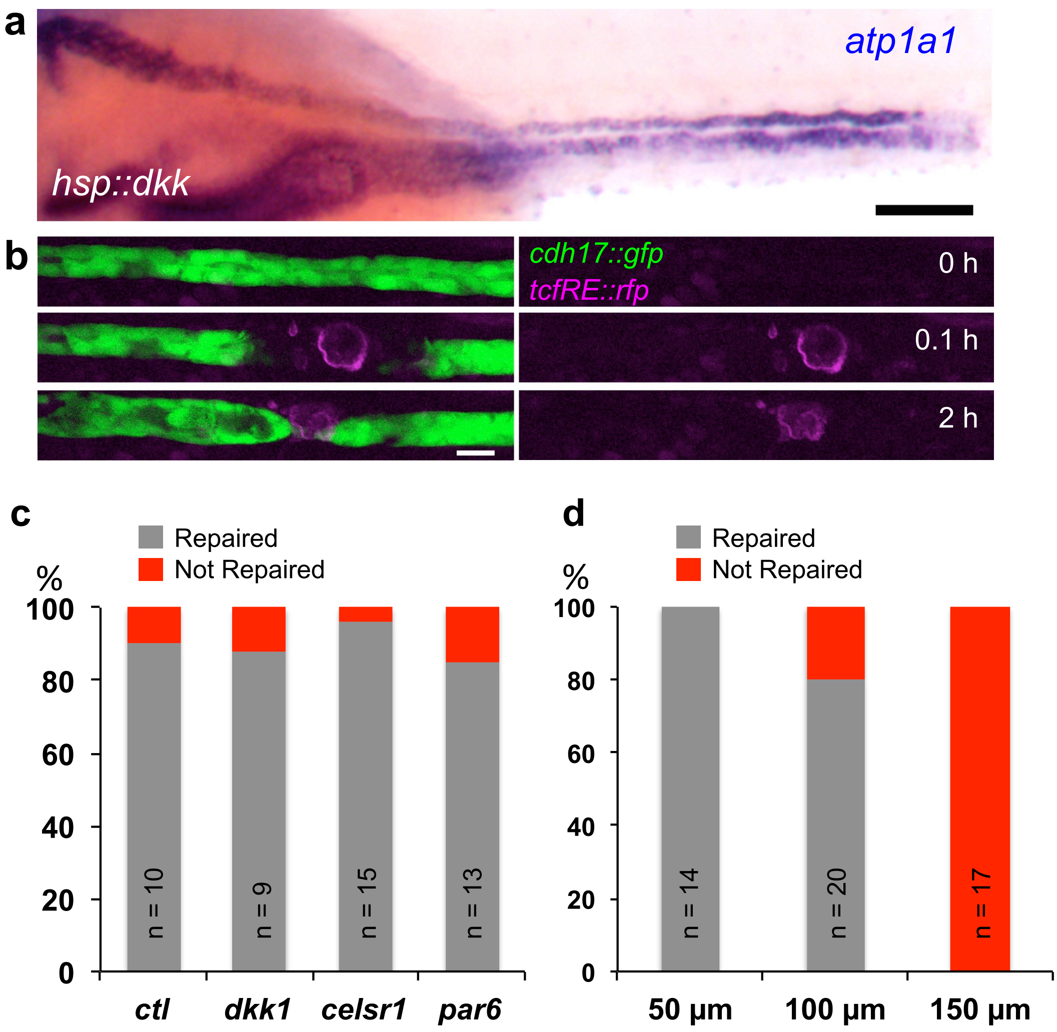

Supplementary Figure 4 | Pronephros repair is independent of Wnt, Celsr1 and Par6 signaling.

(a) *In situ* hybridization with the pronephros specific marker *atp1a1a.4*, staining injured zebrafish embryo over-expressing the Wnt inhibitor *dkk1*. The pronephros was injured 2 days after fertilization and fixed 16 hours later. The injured proximal side (arrow) appears dilated, but recovery was not affected by *dkk1* over-expression (Scale bar, 100 µm). **(b)** Frames from a time-lapse movie of injured transgenic embryo carrying a TCF response element, driving the expression of Red Fluorescence Protein (RFP). Laser ablation caused some background fluorescence (red channel) in the injured cells, but no specific RFP expression was detectable in neighboring cells displaying a migratory response (Scale bar, 10 µm). **(c)** Quantification of the repair process in *HSP:dkk1*, *celsr1* morpholino oligonucleotide (MO) and *par6* MO injected embryos that were injured 2 days after fertilization and fixed 16 hours post wounding. While MO-injected embryos displayed duct abnormalities, repair of the pronephros injury was not affected. **(d)** Injuries ≤ 50 µm in length were repaired in 100%, injuries ≤ 100 µm, corresponding to 10-12 cell diameters, were repaired in 80%, while no injuries ≤ 150 µm were repaired.

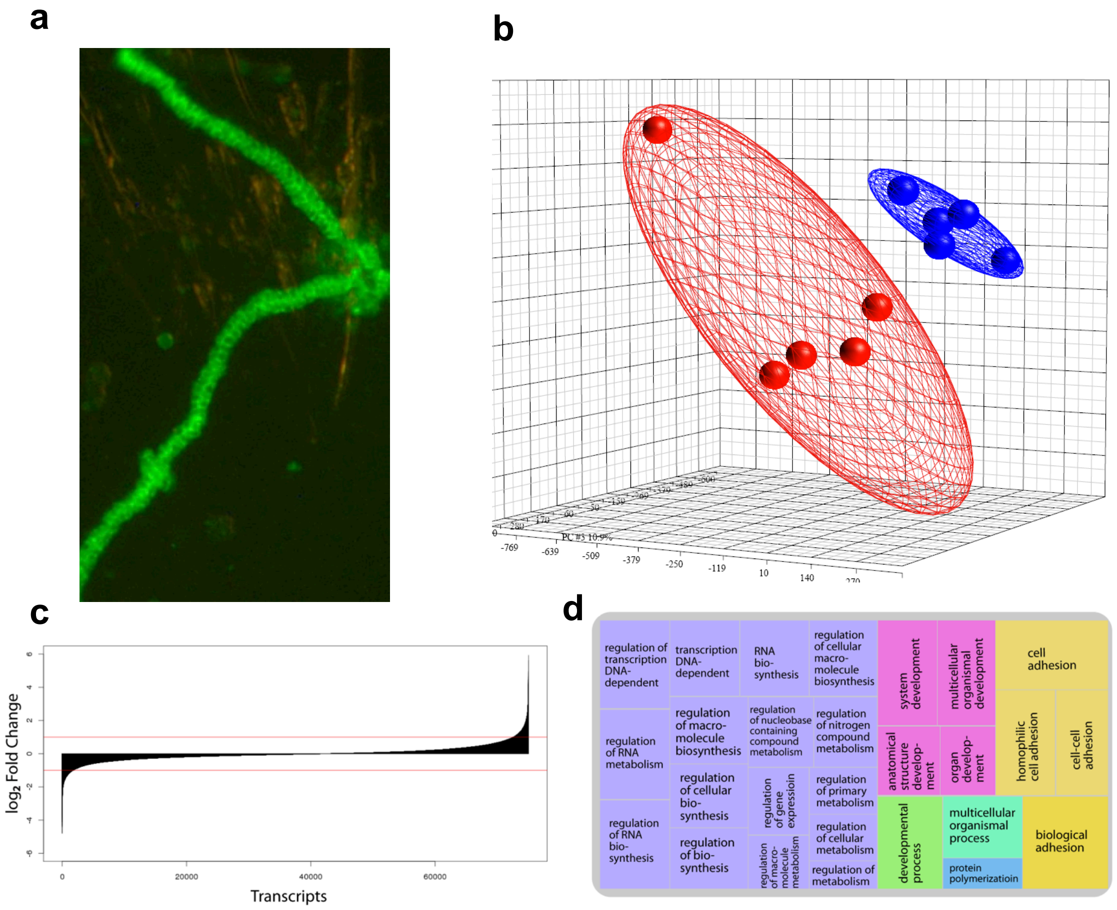

e

d2 vs d1	0-36 h	42-60 h
Up ↑	14	3 (*cxcl12a, myca, mafba*)
down ↓	77	0
No change →	297	17

Supplementary Figure 5 | Transcriptional profiles of one- and two-day-old zebrafish pronephric tubules.
(**a**) Fluorescently labeled pronephric tubules, isolated from *cadh17:gfp* transgenic zebrafish embryos. (**b**) 3D representation of the changes in the total transcript levels between samples from one- (red) and two-day-old zebrafish embryos (blue). Due to technical problems, one of the samples from the first group failed to cluster with the others probes, and was excluded from further analysis. (**c**) Density histogram representing the distribution of the log_2-fold change of all transcripts. The horizontal red lines mark the two-fold change limit. (**d**) Biological process Gene Ontology (GO) terms enrichment analysis of the transcripts that were up-regulated more than two-fold in the pronephros on the second day of development. Semantically similar categories are represented with similar colors. The size of each box reflects the significance of enrichment ($-log_{10}$ p-value). The GO analysis was performed with AmiGO and the GO enrichment was graphically represented with REViGO. (**e**) Comparison with the ZFIN Database and expression profiles, focusing on genes that are specifically expressed in the zebrafish pronephros at 42-60 hours post fertilization, revealed *cxcl12a*, *myca* and *mafba* as potential candidates involved in pronephros repair.

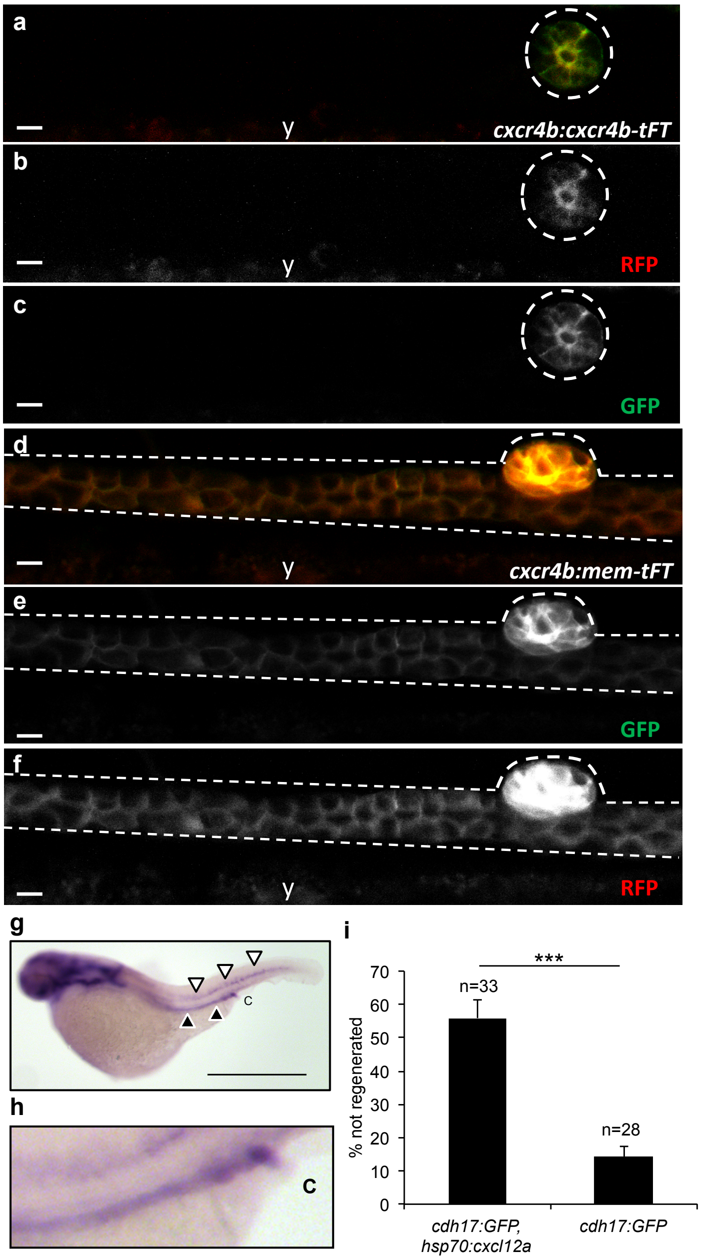

Supplementary Figure 6 | Low-level expression of *cxcr4b* in pronephric duct cells during normal collective cell migration.

(a-c) Using the *cxcr4b:cxcr4b-TFT* (GFP = short life-time, RFP = long life-time) zebrafish line, lifetime Cxcr4b was only detectable in the corpuscle of Stannius (outlined) at 48 hpf, but not in the pronephros. **(d-f)** The membrane-resident version *cxcr4b:mem-TFT,* which does not respond to Cxcl12a signaling and therefore accumulates at the plasma membrane, was detectable in the pronephros (y, yolk) (Scale bars, 10 µm). **(g)** *In situ* hybridization depicts expression of *cxcl12a* in the posterior lateral line (white arrowheads) and in the pronephros (black arrowheads) in zebrafish embryos at 2 dpf (Scale bar, 800 µm). A magnification of the posterior pronephric duct is shown in **(h)** (c, cloaca). **(i)** Ectopic expression of Cxcl12a, triggered by heat-shock in the double transgenic *cdh17:GFP; hsp-70:cxcl12a* zebrafish line, significantly reduces the ability to repair a pronephros injury (means ± SEM; ***, $p < 0.001$; t-test) .

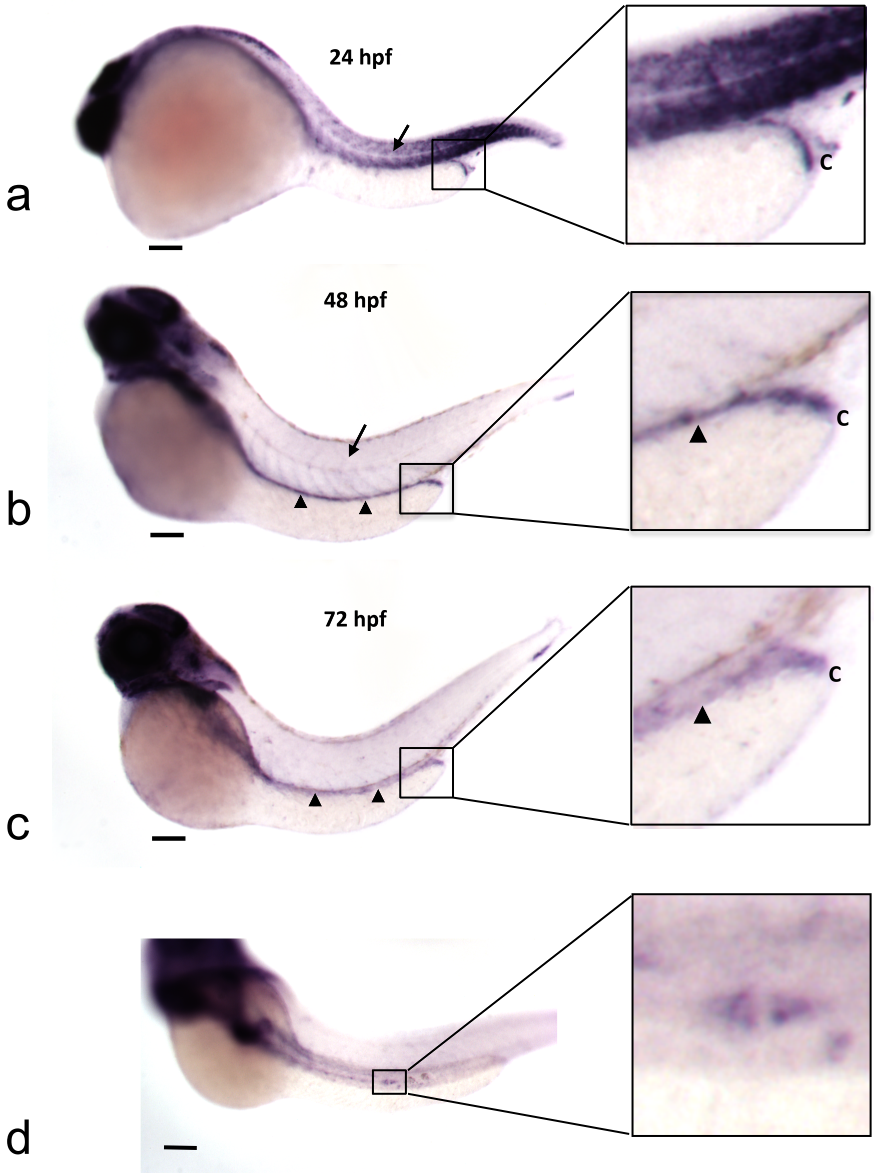

Supplementary Figure 7 | *myca* expression in zebrafish embryos at 1-3 dpf, and up-regulation of *myca* in response to injury.
(a) *myca* was detected by *in situ* hybridization at 24 hpf in the zebrafish pronephros. Insert shows the posterior pronephros region close to the cloaca (c, cloaca). *myca* was strongly expressed in the head, the myotomes (arrow) and in the cloaca (magnification, c) at this stage. **(b)** Expression of *myca* was restricted to the head, the somatic furrow (arrow) and the pronephros (arrowheads) at 48 hpf. **(c)** Expression of *myca* in the somitic furrows declined at 72 hpf, but remained stable in the head, pronephros (arrowheads) and in the cloaca (c). **(d)** *myca* was up-regulated in the pronephric duct cells adjacent to a laser-induced wound at 48 hpf (magnification; c, cloaca) (Scale bars, 50 µm).

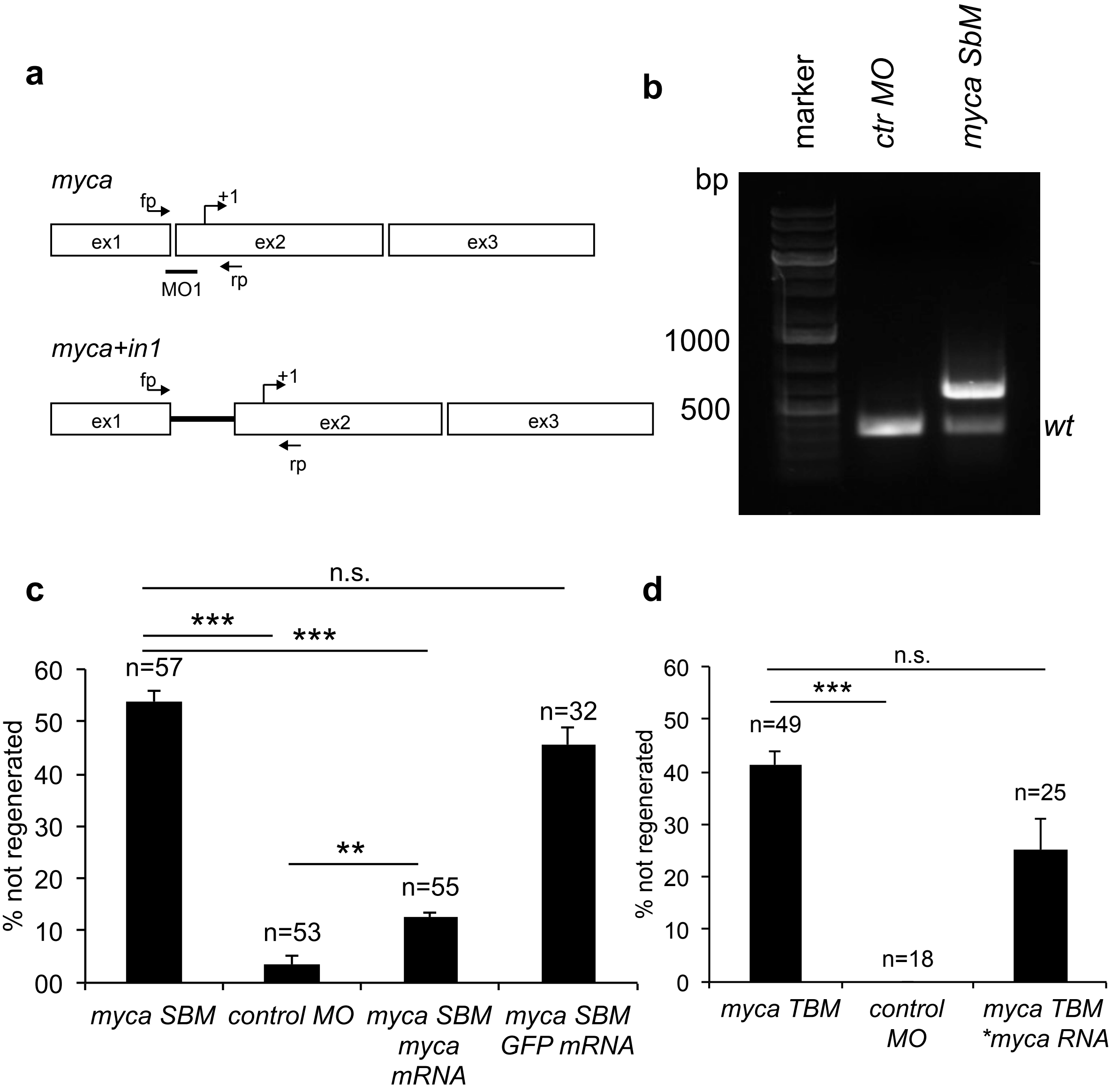

Supplementary Figure 8 | Splice-blocking morpholino oligonucleotide (SBM) directed against *myca* results in intron inclusion.

(a) The splice-blocking morpholino oligonucleotide (SBM) directed against *myca* targets the intron1-exon 2 junction and results in intron inclusion (*myca+in1*), and leads to a STOP codon shortly after. **(b)** The intron inclusion event is detectable by PCR in *myca* SBM injected embryos as a higher molecular weight band (*myca+in1*) compared to control MO injected embryos. **(c)** While *myca* mRNA partially rescued the repair defect caused by *myca* SBM MO, an inactive *GFP* mRNA (200 ng/µL) did not significantly reduce the repair defect. **(d)** Mutant **myca* mRNA (150 ng/µL), containing four nucleotide substitutions to prevent binding of the translation-blocking morpholino oligonucleotide (TBM), did not significantly rescue the repair defect caused by the *myca* translation-blocking MO (TBM) (mean ± SEM; **, $p < 0.01$; ***, $p < 0.001$; n.s., not significant; t-test).

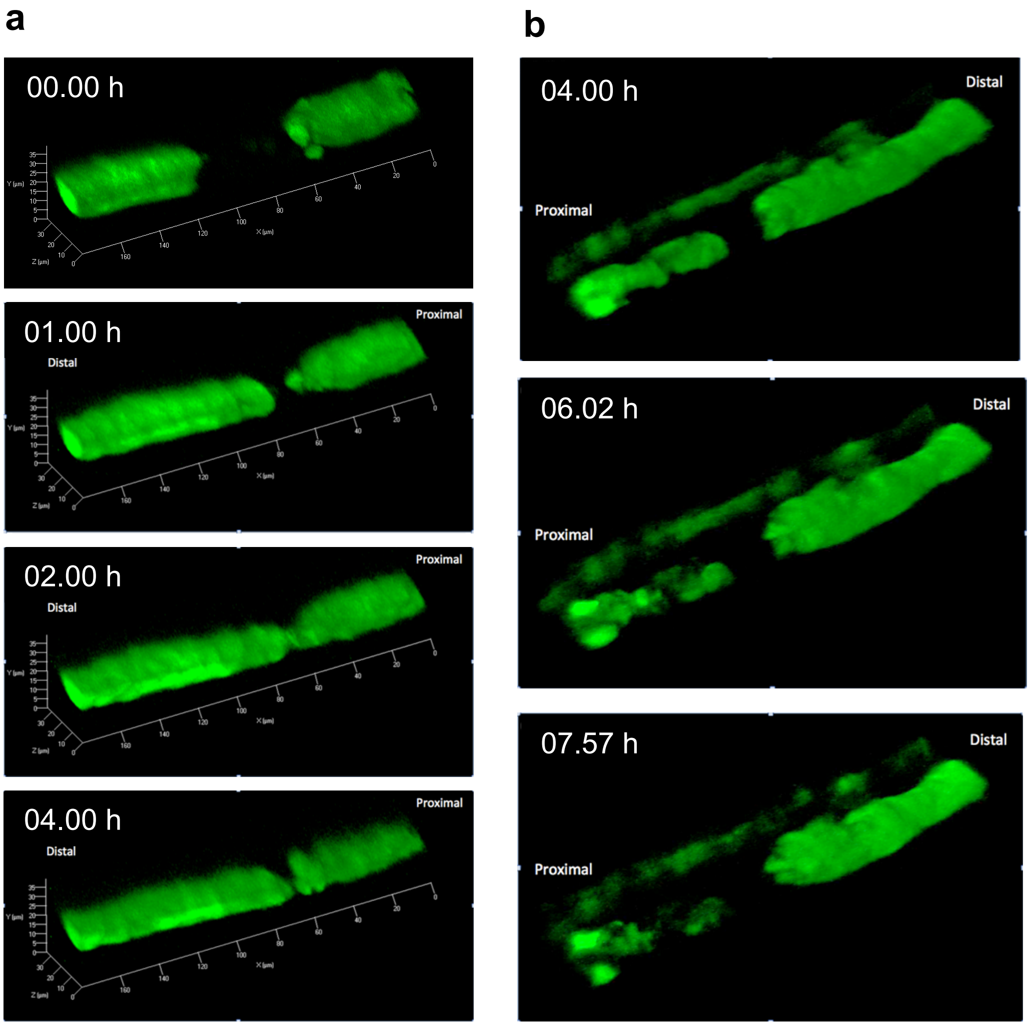

Supplementary Figure 9 | Zebrafish *myca* depletion causes a repair defect characterized by abnormal migratory behavior.
(a) Single frames taken from the Supplementary Movie 8. Depletion of *myca* prevented the reversal of the posterior-to-anterior cell migration at the proximal side of the injury, resulting in a failure to repair the injury. **(b)** Single frames taken from the Supplementary Movie 9. Depletion of *myca* prevented the repair within 8 hours after a laser-induced injury. Instead of rapidly repairing the injury by a migratory response, the *myca*-deficient pronephric duct cells remained stationary.

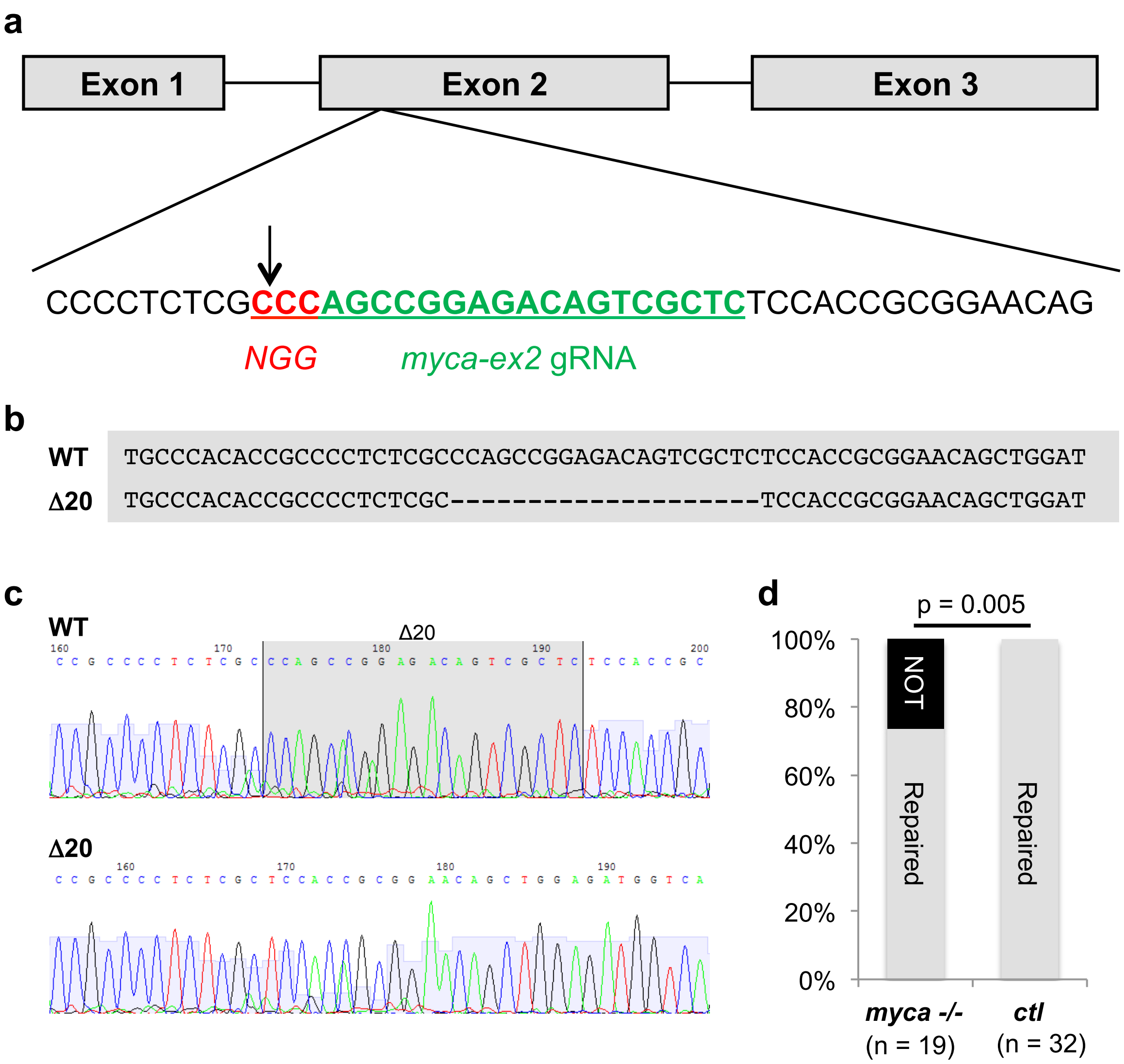

Supplementary Figure 10 | A zebrafish *myca* mutant displays a repair defect. (**a**) The depicted *myca-ex2* gRNA (*NGG*, canonical protospacer adjacent motif (PAM) sequence) was used to induce a 20-bp deletion in the second exon of *myca*. F0 founders were crossed with *wt1b:GFP; cdh17:GFP* transgenic zebrafish. F1 heterozygous fish, which carried the 20-bp deletion, were outcrossed to establish a heterozygous line. The F2 heterozygous animals were in-crossed to generate homozygous *myca* Δ20 zebrafish embryos. (**b**) Alignment between wild-type and mutant *myca* depicts the 20-bp deletion within the second exon. (**c**) Sequencing results of wild-type and the *myca* Δ20mutant zebrafish line. **(d)** Heterozygous myca$^{+/-}$ zebrafish were crossed, and injured two days post fertilization. A total of 51 embryos from three independent experiments were genotyped. While 25% of mutant *myca*$^{-/-}$ zebrafish embryos did not repair, none of the heterozygous *myca*$^{+/-}$ or wild-type *myca*$^{+/+}$ (*ctl,* controls) revealed a repair defect (Fischer's exact test, two-tailed p value).

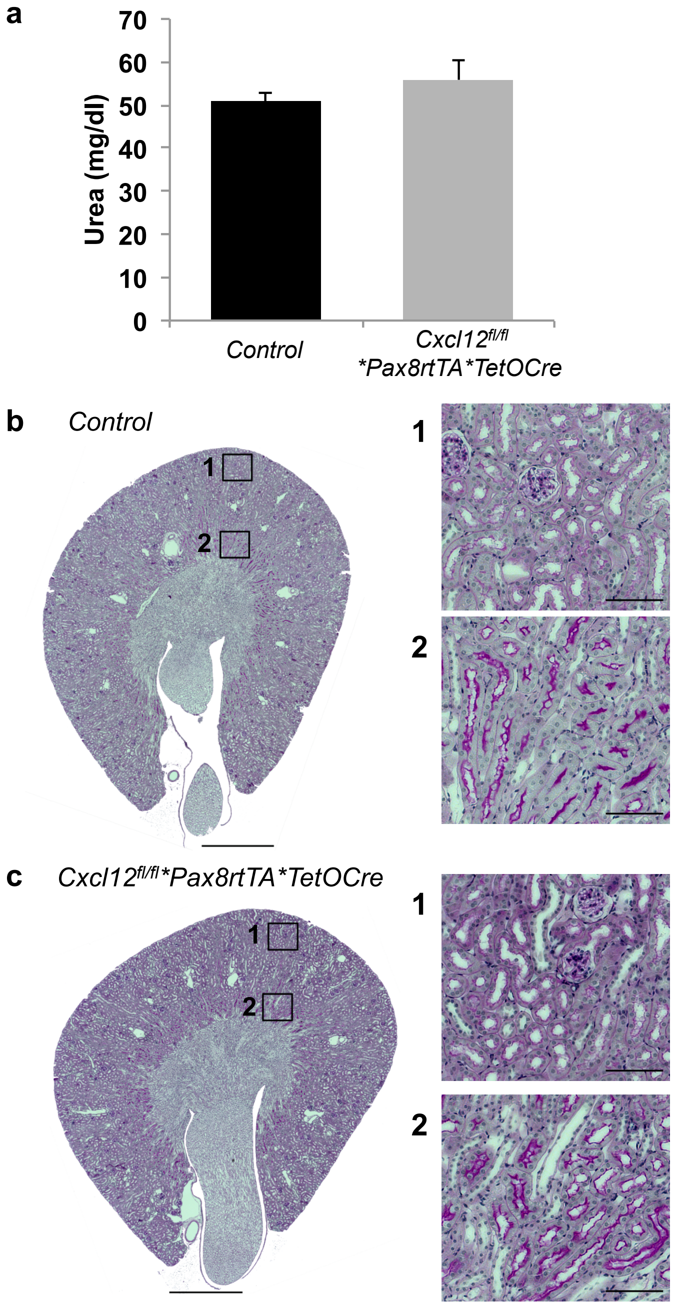

Supplementary Figure 11 | Kidney-specific elimination of *Cxcl12* does not impair renal function at baseline.

(**a**) Male mice at the age between 10-12 weeks with the genotype *Cxcl12$^{fl/fl}$*Pax8rtTA*TetOCre* (n=5) and control mice (n=5) with the genotype *Cxcl12$^{fl/fl}$*Pax8rtTA* were treated with doxycycline (2 mg/mL for 2 weeks) to induce the renal tubule-specific excision of *Cxcl12*, followed by a doxycycline wash-out period of one week. There was no difference in urea levels immediately before the ischemia/reperfusion injury (mean ± SEM; t-test). (**b**) and (**c**) Comparison of tissue sections from control and *Cxcl12$^{fl/fl}$*Pax8rtTA*TetOCre* kidneys showed a normal nephron architecture (1, magnification of the square in the cortical region, 2, magnification of the square in the medullary region) (Scale bars, 1 mm and 100 μm).

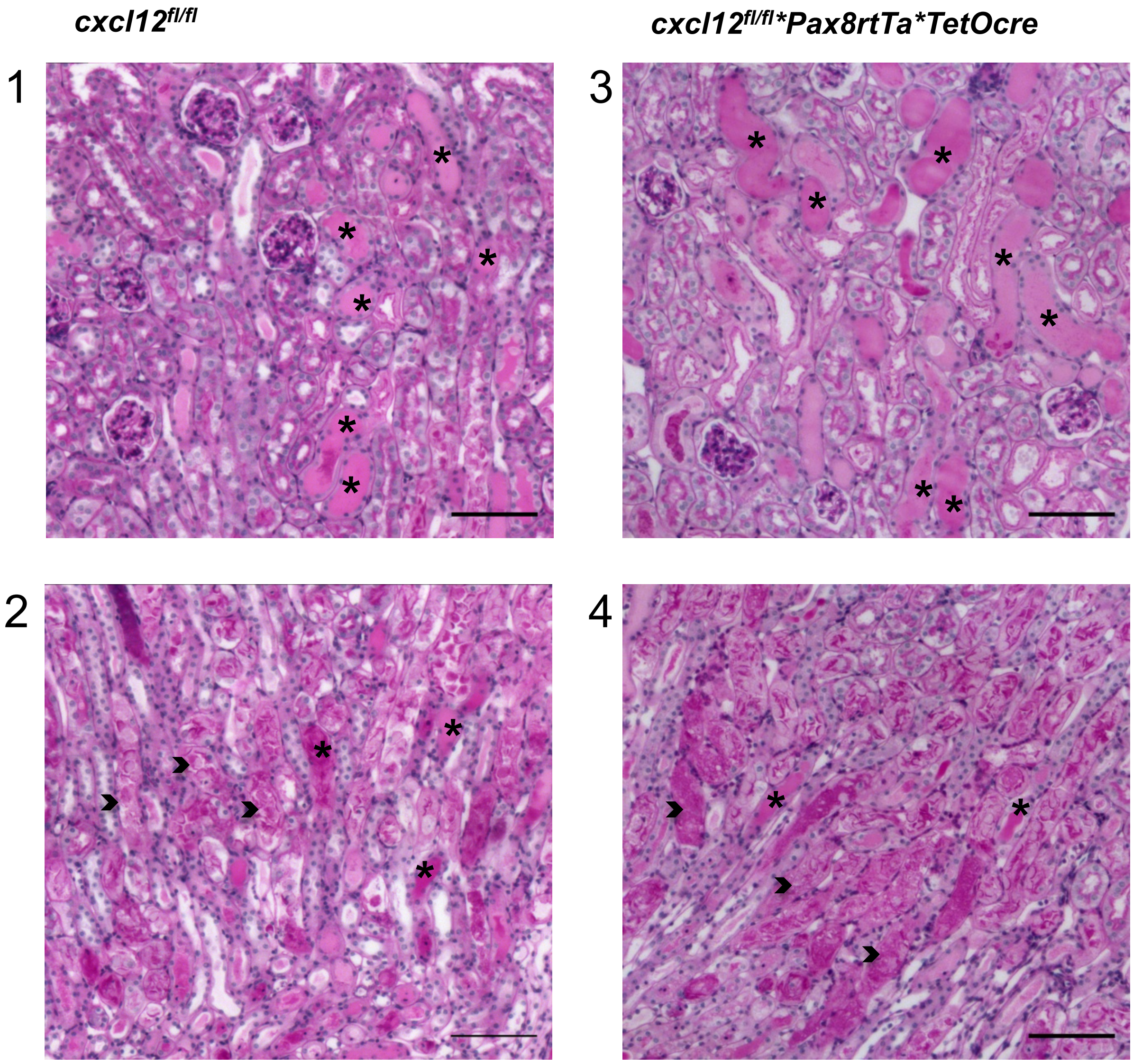

Supplementary Figure 12 | Kidney-specific elimination of *Cxcl12* impairs the recovery 12 hours after ischemia/reperfusion injury.

High-resolution micrographs are depicted that correspond to Figure 6d (1-4). Arrowheads and stars highlight intraluminal debris and casts, respectively (Scale bars, 100 µm).

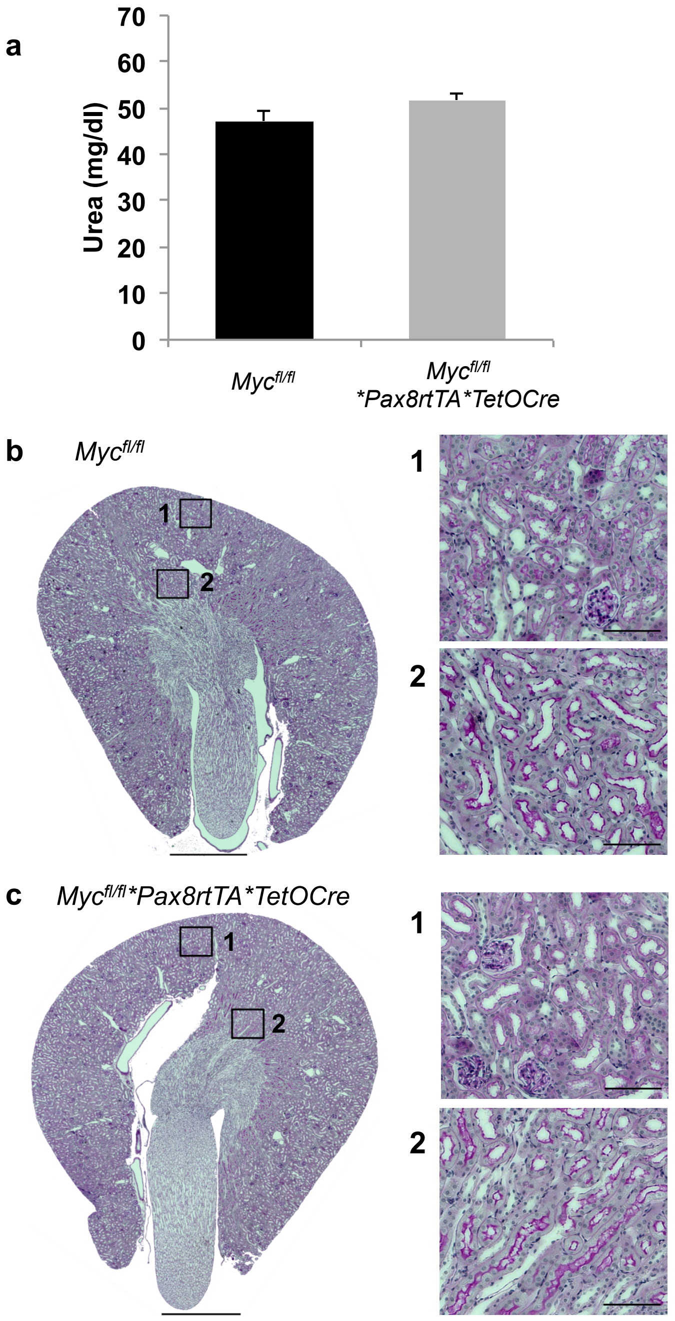

Supplementary Figure 13 | Kidney-specific elimination of *Myc* does not impair renal function at baseline.

(**a**) Male mice at the age between 10-12 weeks with the genotype *Myc*$^{fl/fl}$**Pax8rtTA*TetOCre* (n=6) and control mice (n=4) with the genotype *Myc*$^{fl/fl}$**Pax8rtTA* were treated with doxycycline (2 mg/mL for 2 weeks) to induce the renal tubule-specific excision of *Cxcl12*, followed by a doxycycline wash-out period of one week. There was no difference in urea levels immediately before the ischemia/ reperfusion injury (mean ± SEM; t-test). (**b**) and (**c**) Comparison of tissue sections from control and *Myc*$^{fl/fl}$**Pax8rtTA*TetOCre* kidneys showed a normal nephron architecture (1, magnification of the square in the cortical region, 2, magnification of the square in the medullary region) (Scale bars, 1 mm and 100 μm).

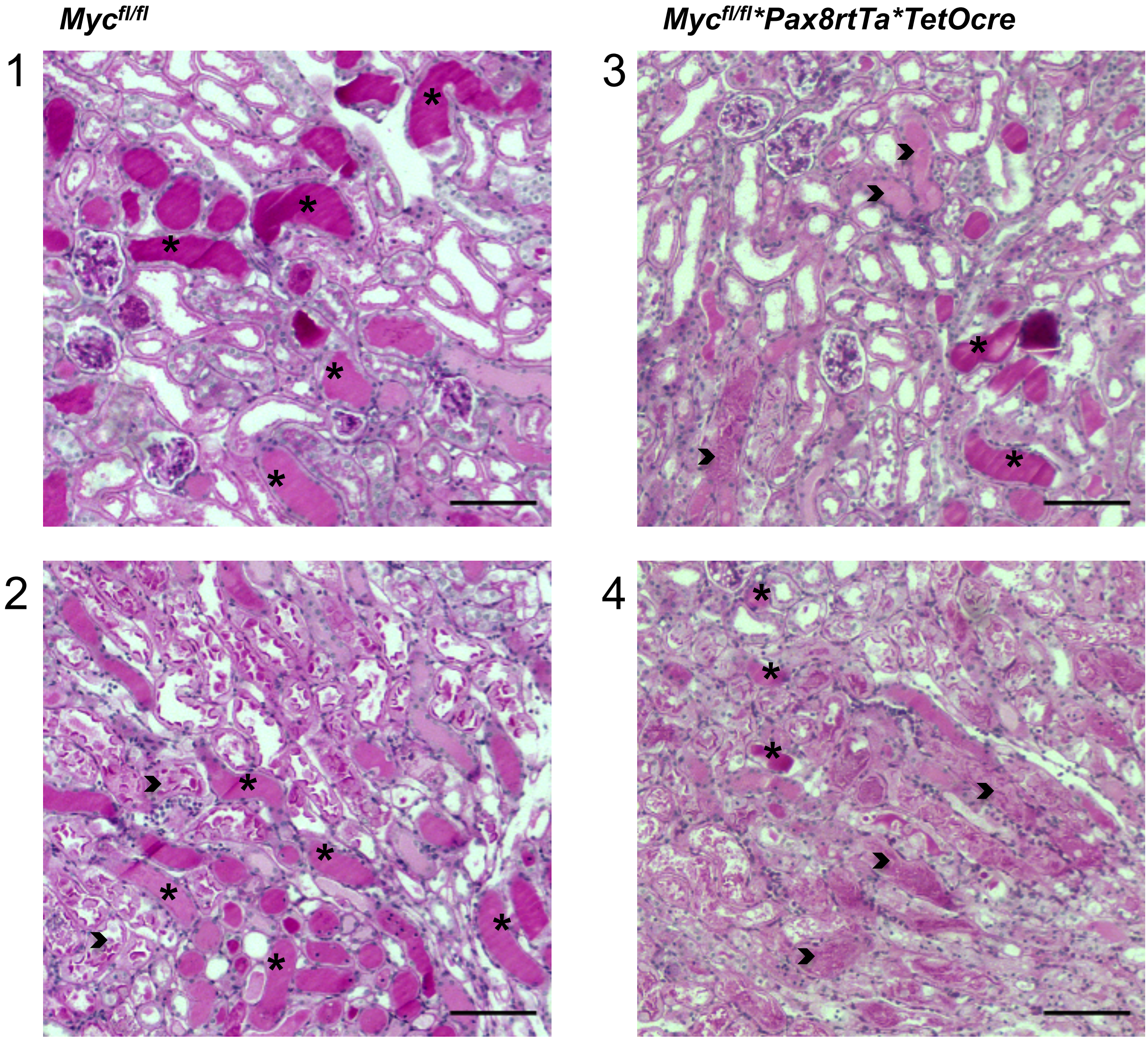

Supplementary Figure 14 | Kidney-specific elimination of *Myc* impairs the recovery 12 hours after ischemia/reperfusion injury.

High-resolution micrographs are depicted that correspond to Figure 6h (1-4). Arrowheads and stars highlight intraluminal debris and casts, respectively (Scale bars, 100 µm).

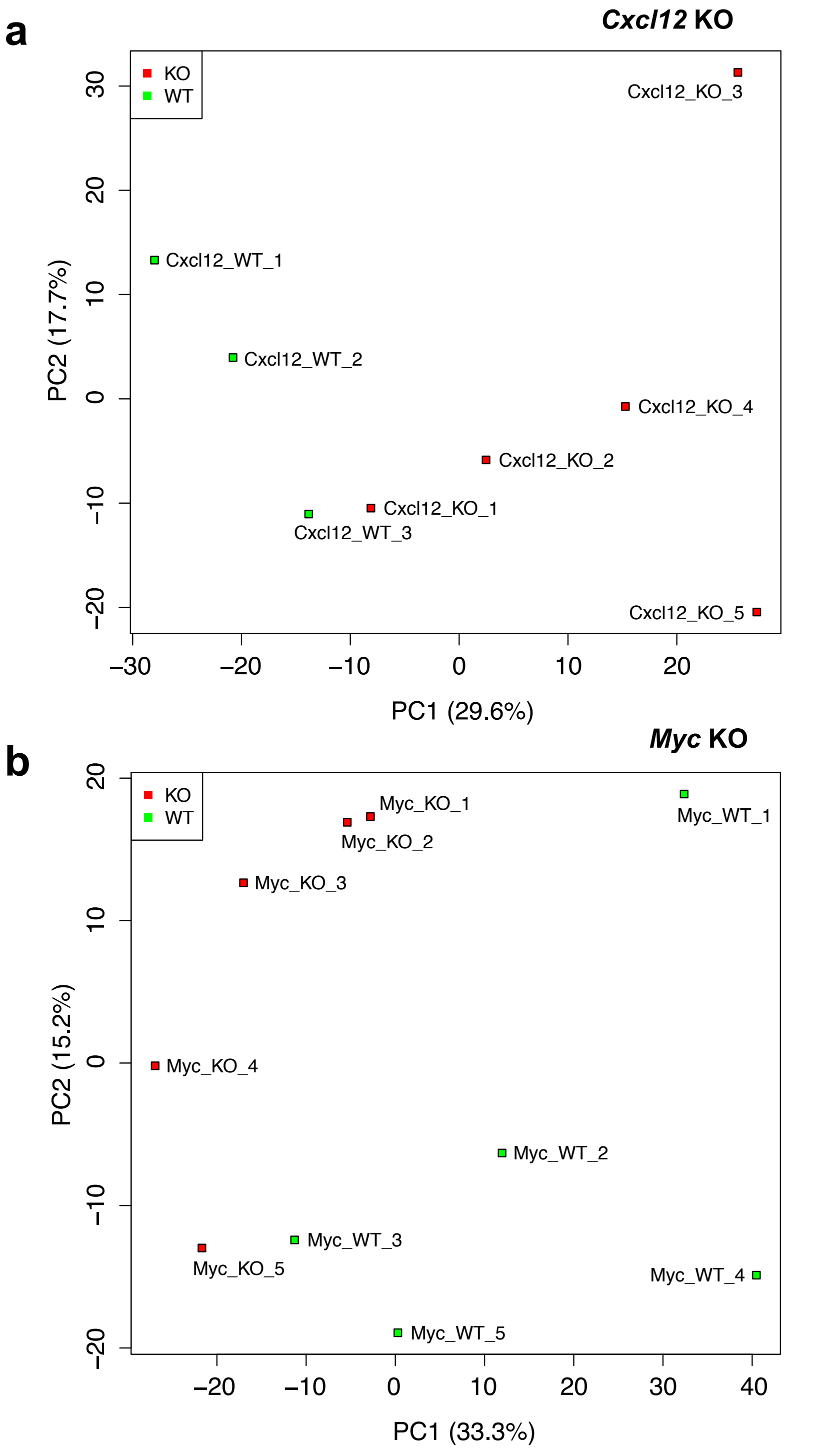

Supplementary Figure 15 | Differential gene expression between control and *Cxcl12* KO or *Myc* KO mice.

Principal component analysis (PCA) segregates control mice and mice with (**a**) kidney tubule-specific excision of *Cxcl12* (*Cxcl12*$^{fl/fl}$**Pax8rtTA*TetOCre*) (*Cxcl12* KO), or (**b**) kidney tubule-specific excision of *Myc* (*Myc*$^{fl/fl}$**Pax8rtTA*TetOCre)* (*Myc* KO).

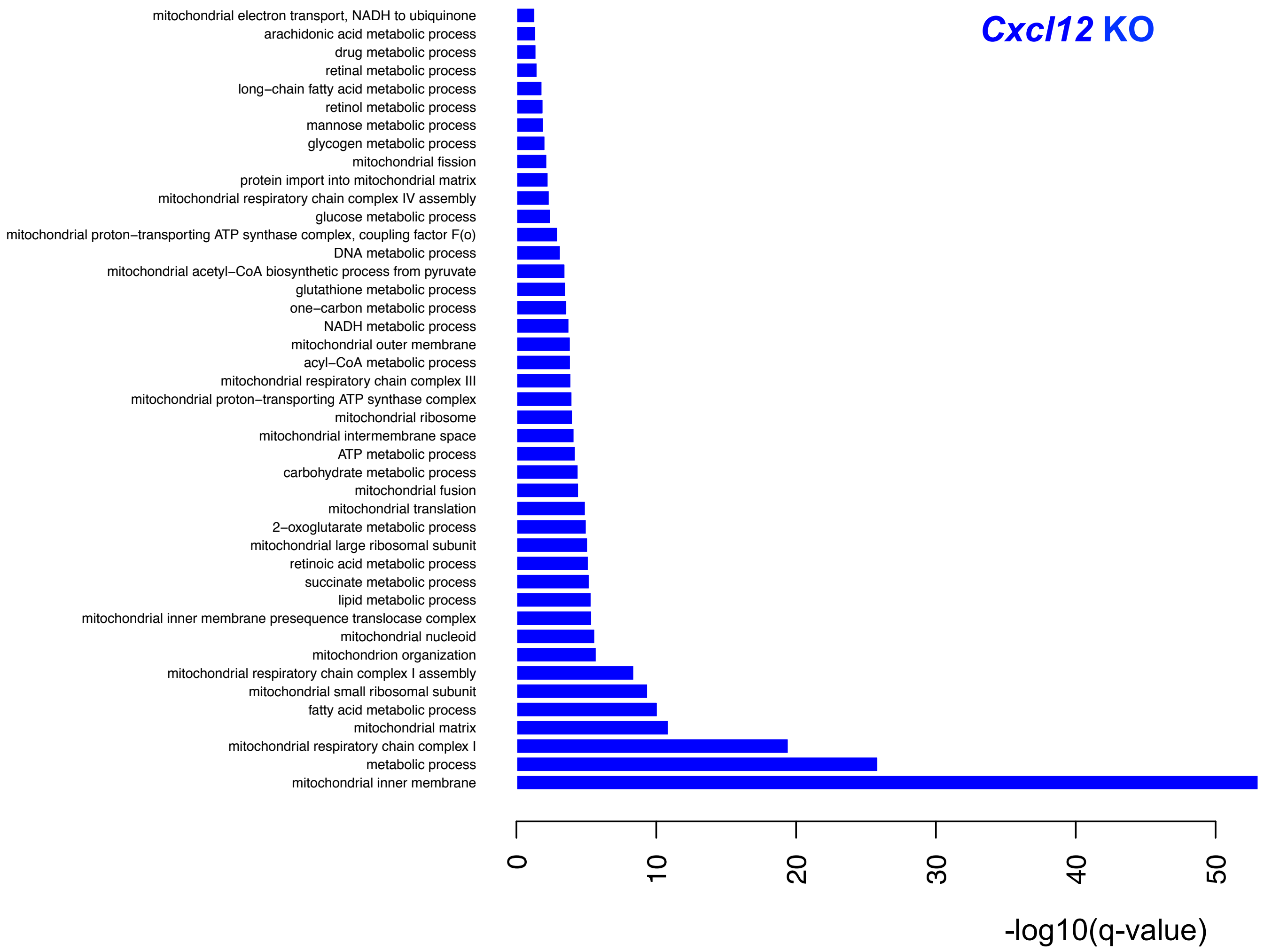

Supplementary Figure 16 | Down-regulation of GO terms involved in mitochondrial- and metabolism-associated processes in *Cxcl12* KO mice. Comparison of control and KO mice exposed to ischemia/reperfusion injury reveals significantly down-regulation of GO terms involved in mitochondrial- and metabolism-associated processes in both KO mice.

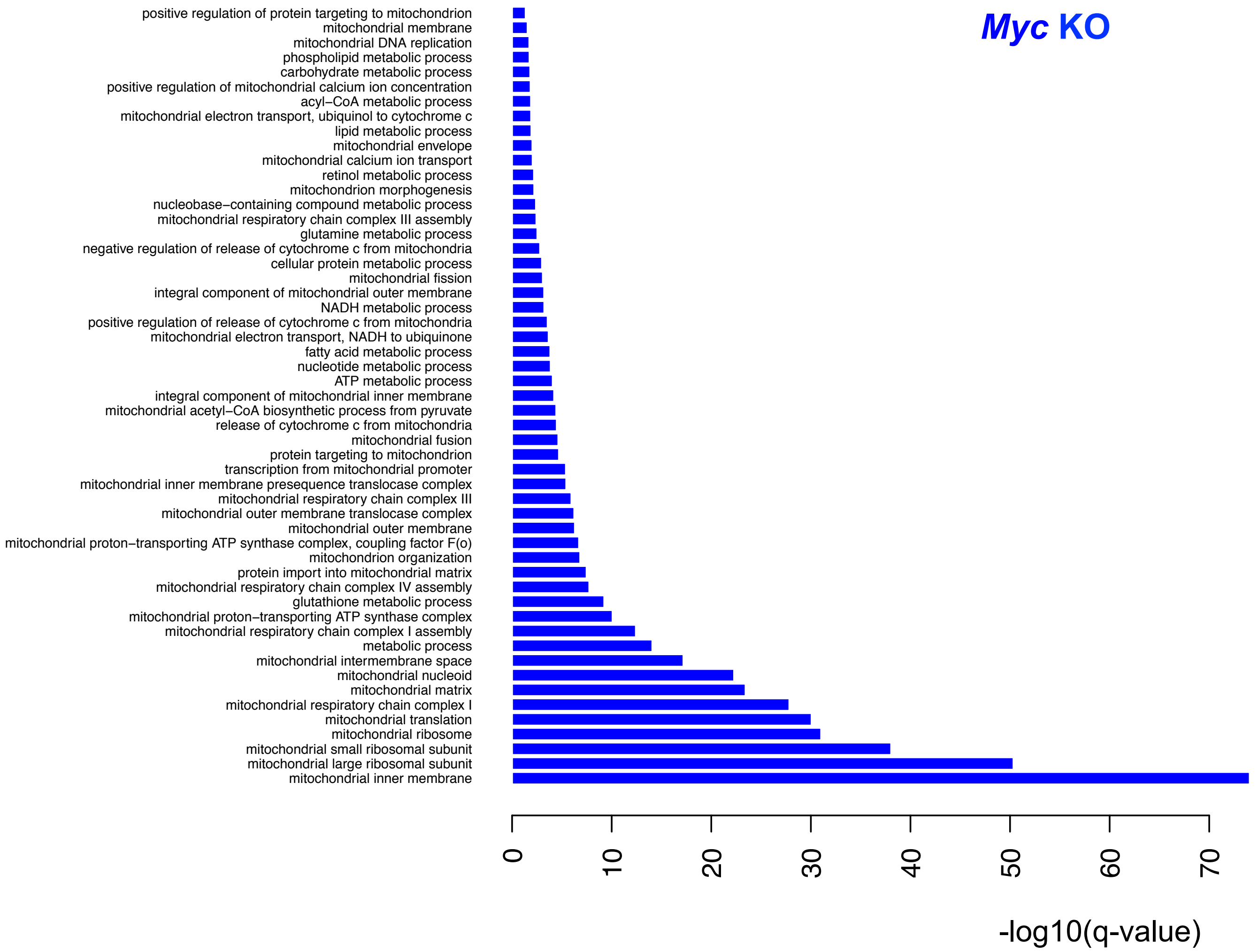

Supplementary Figure 17 | Down-regulation of GO terms involved in mitochondrial- and metabolism-associated processes in *Myc* KO mice. Comparison of control and KO mice exposed to ischemia/reperfusion injury reveals significantly down-regulation of GO terms involved in mitochondrial- and metabolism-associated processes in both KO mice.

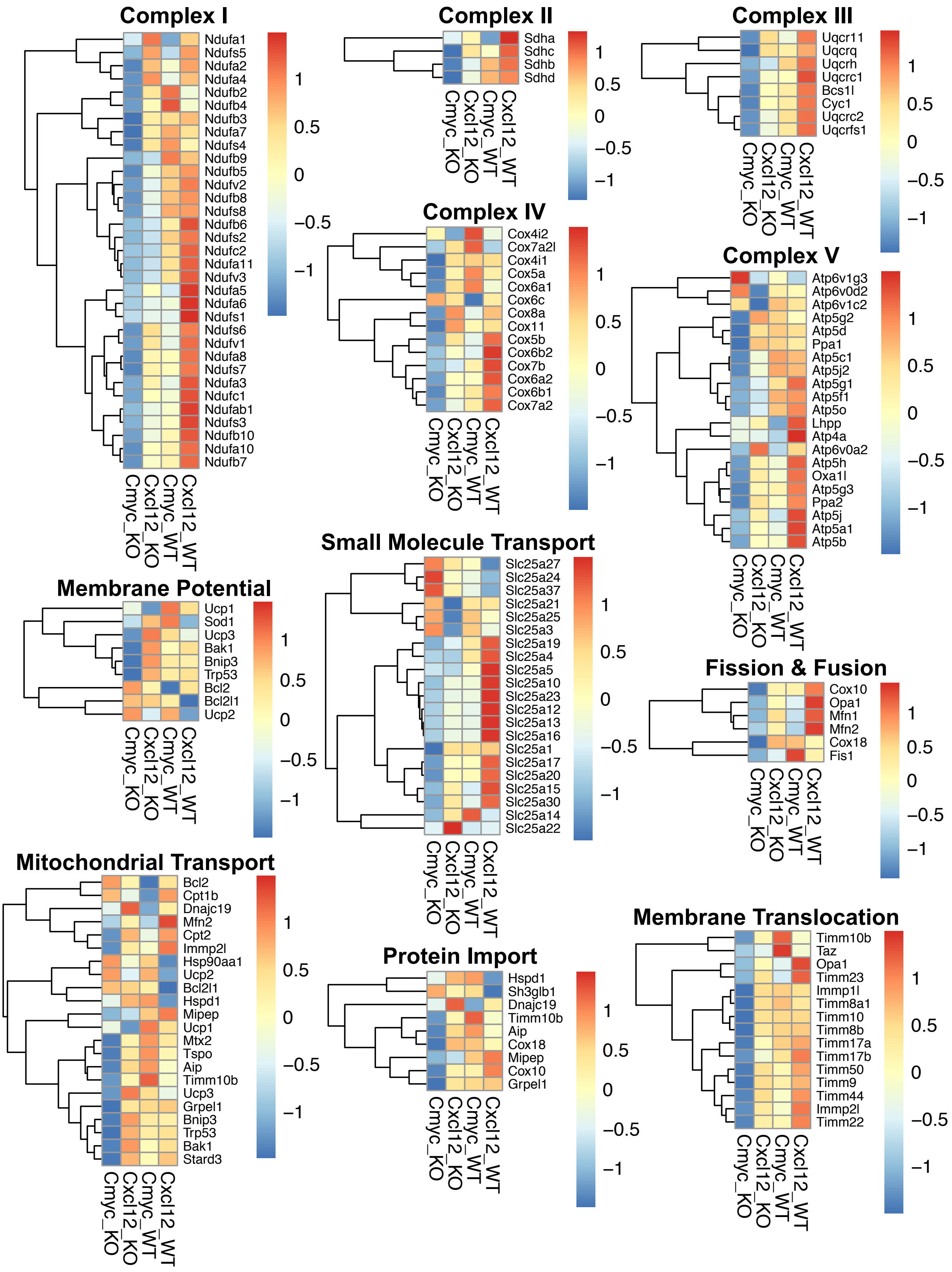

Supplementary Figure 18 | Heat-maps depicting the abnormalities in mitochondrial functions in *Cxcl12* and *Myc*-deficient mice. Mice were grouped according to their genotype (*Cxcl12_WT, Myc_WT, Cxcl12_KO, Myc_KO*). Up- and down-regulation of genes are color-coded with red depicting up-regulated genes, and blue representing down-regulated genes.

a ***Cxcl12* KO: Top 25 down-regulated GO terms**

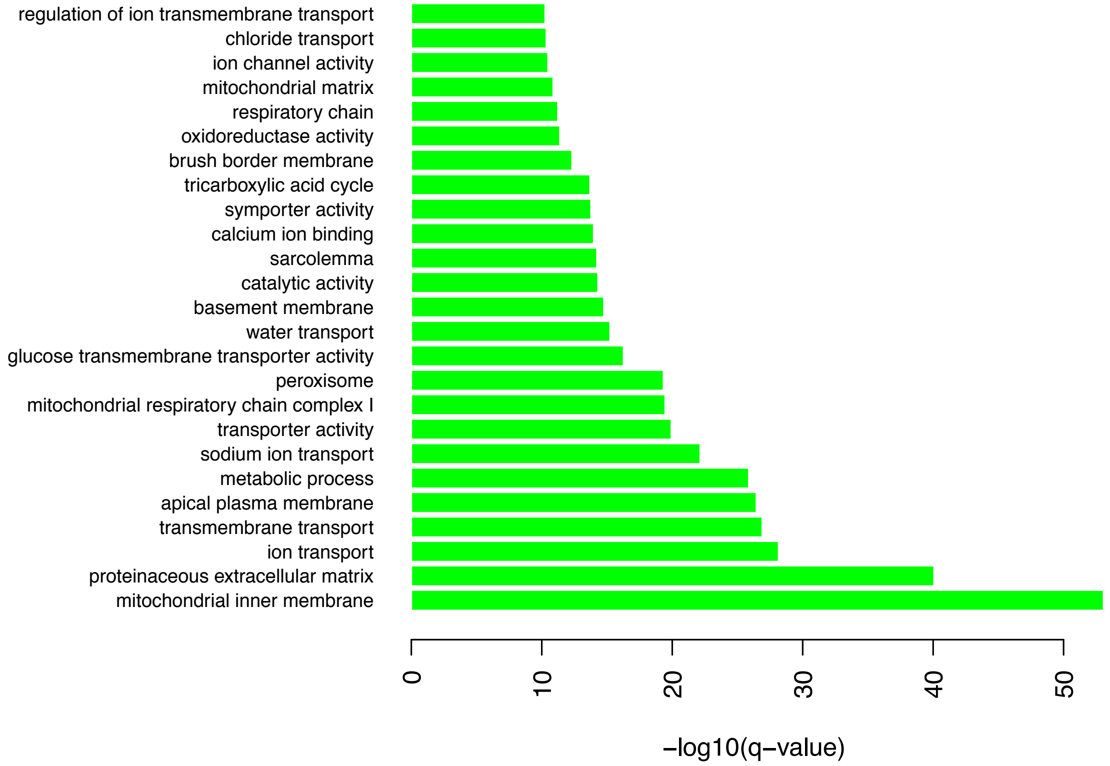

b ***Myc* KO: Top 25 down-regulated GO terms**

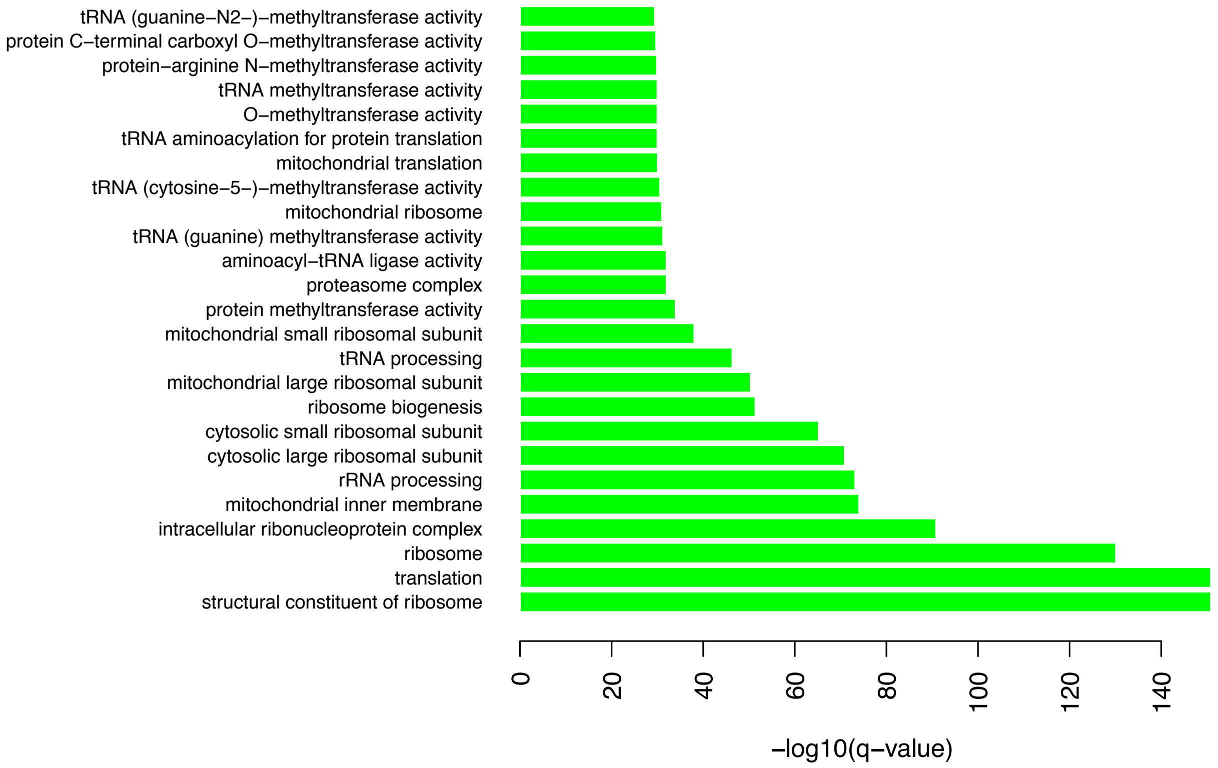

Supplementary Figure 19 | Top 25 down-regulated GO terms in *Cxcl12* and *Myc* KO mice. GESA of GO term analysis was performed comparing gene expression profiles of control and KO mice exposed to ischemia/reperfusion injury.

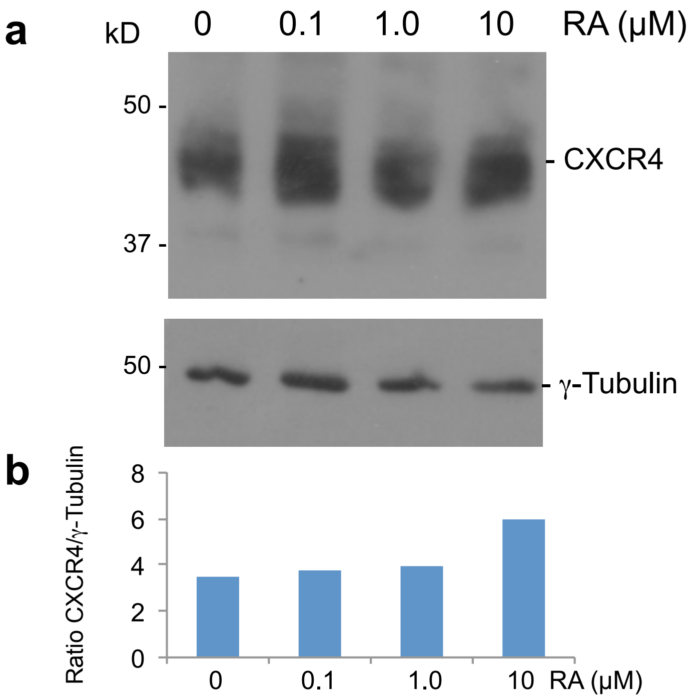

Supplementary Figure 20 | CXCR4 is induced by tretinoin in Jurkat cells.
(a) Jurkat cells were stimulated with tretinoin (RA) in concentrations between 0.1 and 10 µM for 24 hours. Western blot analysis was performed for CXCR4 and γ-tubulin. **(b)** CXCR4 levels were normalized for γ-tubulin levels, demonstrating an increase in CXCR4. Representative images from 3 independent experiments.

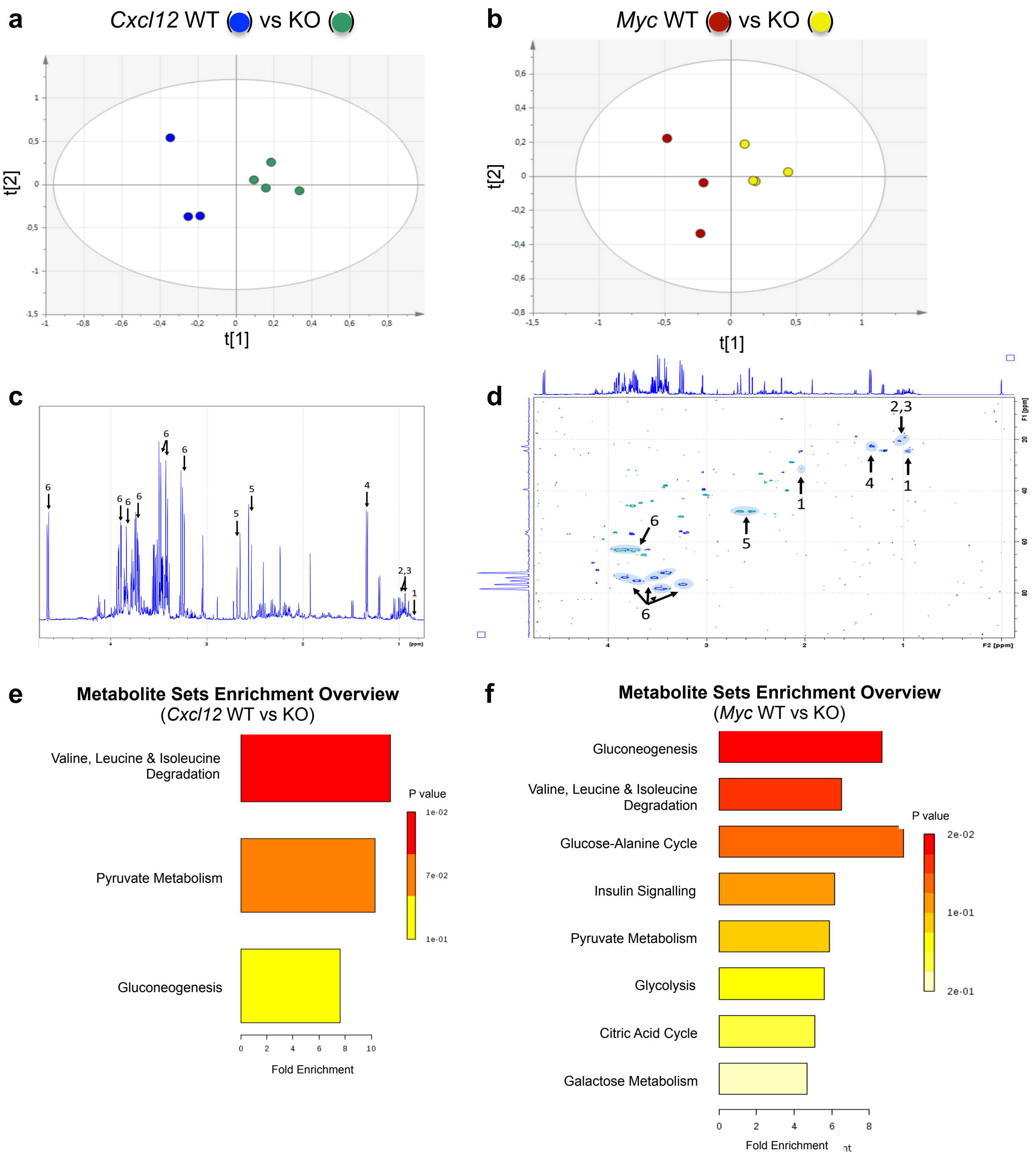

Supplementary Figure 21 | Urinary metabolites suggest abnormal glucose metabolism in *Cxcl12*- and *Myc*-deficient mice.

Score plot from PLS-DA applied to 1H-NMR spectra of **(a)** control versus *Cxcl12* KO (2A, R^2=0,927, Q^2=0,343) and **(b)** control versus *Myc* KO (2B, R^2=0,878, Q^2=0,265) mouse urine following I/R injury. The principal component analysis (PCA) identified two outliers in the *Myc* condition (one wild-type and one *Myc* KO urine) that were excluded from further analysis. **(c)** Representative 1H-NMR and **(d)** Heteronuclear Single-Quantum Correlation (HSQC) spectra of *Cxcl12* KO mouse urine. Relevant metabolites are numbered (1: α-hydroxy-isovalerate, 2,3: leucine, isoleucine, 4: lactate, 5: citrate, 6: glucose). Identification (via Metabolite Set Enrichment Analysis) of pathways affected by **(e)** *Cxcl12* KO, or **(f)** *Myc* KO.

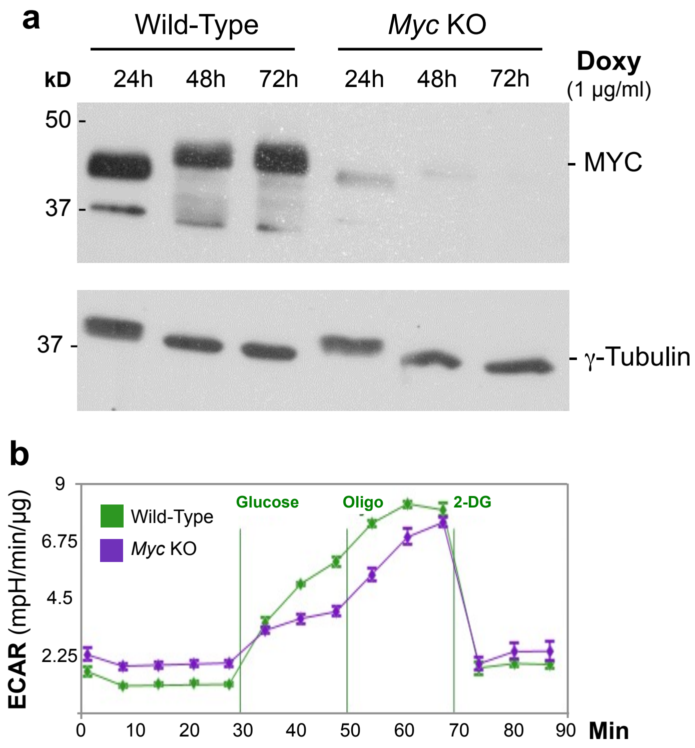

Supplementary Figure 22 | Defective glycolytic capacity in *Myc*-deficient tubular epithelial cells.

(a) Tubular epithelial cells were isolated from wild-type and *Mycfl/fl*Pax8rtTA*TetOCre* mice. After incubation with doxycycline (1.0 µg/ml) for 24 hours, MYC was almost completely depleted. Representative images from 3 independent experiments. **(b)** For ECAR measurements, wild-type and *Mycfl/fl*Pax8rtTA*TetOCre* renal epithelial cells were treated with doxycycline (0.5 µg/ml) for 24 hours, followed by a 24-hour incubation period without doxycycline to avoid doxycycline-dependent metabolic effects. Cells were then exposed to glucose-free XF24 Seahorse medium for one hour, followed by sequential addition of glucose (10 mM), oligomycin (Oligo) (4 µM) and 2-deoxy-D-glucose (2-DG) (50 mM). ECAR was normalized to the protein concentration in each well, revealing a decreased maximal glycolytic capacity in *Myc*-deficient tubular epithelial cells. Measurements were performed in triplicates, and depicted as means ± SD.

a

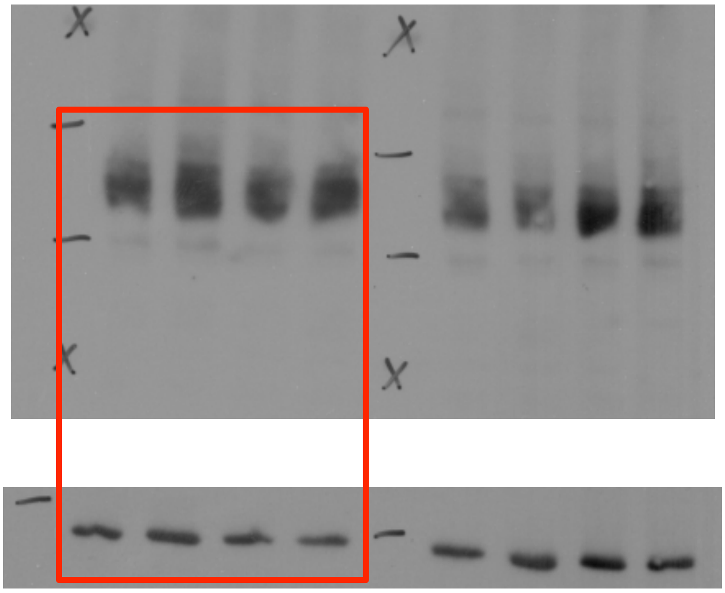

b

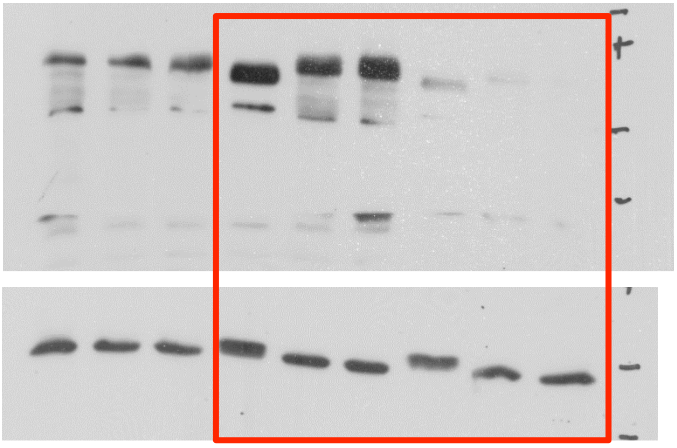

Supplementary Figure 23 | Uncropped Western blots.
(a) Western blot depicted in Supplementary Figure 20.
(b) Western blot depicted in Supplementary Figure 22a.
