# Supplementary material for: CXCL12 and MYC control energy metabolism to support adaptive responses after kidney injury
Source: Nat Commun. 2018 Sep 10;9:3660. doi: 10.1038/s41467-018-06094-4 (PMC6131511; doi:10.1038/s41467-018-06094-4)
Supplement: Supplementary file 3 — Description of Additional Supplementary Files [file 41467_2018_6094_MOESM3_ESM.docx]

**Description of Additional Supplementary Files**

File Name: Supplementary Data 1

Description: Up-regulated gene ontology (GO) enrichment terms, comparing Cxcl12 KO and control mice.

File Name: Supplementary Data 2

Description: Down-regulated gene ontology (GO) enrichment terms, comparing Cxcl12 KO and control mice.

File Name: Supplementary Data 3

Description: Up-regulated gene ontology (GO) enrichment terms, comparing Myc KO and control mice.

File Name: Supplementary Data 4

Description: Down-regulated gene ontology (GO) enrichment terms, comparing Myc KO and control mice.

File Name: Supplementary Movie 1

Description: Repair of a laser-induced injury by cell migration. Neighboring tubular epithelial cells migrate to repair the injured pronephros. Part of the pronephric duct of a two-day-old *NaK-atp1a1a.4:mCherry* transgenic embryo was ablated using a two-photon laser. The region was imaged over four hours using confocal microscopy. A summary of the direction and net track displacement is shown with green arrows. The non-injured cells flanking the gap migrate actively towards each other to close the gap caused by cell ablation. Note that cells anterior to the injury (left) reverse the direction of the posterior-to-anterior collective cell migration normally observed in two-day-old embryos.

File Name: Supplementary Movie 2

Description: Dual injuries of the pronephric duct of a two-day-old zebrafish embryo. The pronephric duct of a *NaK-atp1a1a.4:mCherry* transgenic embryo was simultaneously injured at two positions at 2 dpf, isolating a patch of cells form the rest of the duct. The region was imaged over four hours using confocal microscopy. The cells adjacent to the injury migrated towards each other until the integrity of the duct was reestablished. The flanking cells of the isolated patch migrated in opposite directions exhibiting similar net displacement, causing stretching and thinning up of the cells within the center of the patch. The forces exerted by the migrating ends seemed similar in both directions, indicated by the small net displacement of the cells in the center of the patch.

File Name: Supplementary Movie 3

Description: Comparison between collective cell migration and repair-induced migration A two-day-old *NaK-atp1a1a.4:h2b-gfp* transgenic embryo was mounted on the side to image both pronephric ducts simultaneously by confocal microscopy. The proximal (lower) duct was injured with a two-photon laser and the region was imaged over nine hours. While the cells of the non-injured duct as well as the cells posterior to the wounding continue their posterior-to-anterior collective migration, the cells anterior to the injury reverse their migration direction. Cell divisions are occasionally observed in both ducts.

File Name: Supplementary Movie 4

Description: Collective cell migration in *cxcl12a(-/-)* and *cxcr4b(-/-)* zebrafish embryos. Collective cell migration of pronephric tubular epithelium in *cxcr4b:H2B–RFP; cldnb:GFP* zebrafish larvae at 48 hpf shown for a *cxcl12a (+/-)* zebrafish embryo. The membranes of pronephric cells are labeled with GFP. The nuclei of *cxcr4b* expressing cells are labeled with RFP.

File Name: Supplementary Movie 5

Description: Quantification of pronephric collective cell migration The RFP signal from pronephric nuclei was used to generate a model of migration with Imaris. From the model quantitative data of migration was obtained for each track.

File Name: Supplementary Movie 6

Description: Cxcr4b up-regulation in leading cells. After laser ablation in *cxcr4b:cxcr4b-TagRFP-sfGFP; cdh17:GFP* larvae at 48 hpf, accumulation of mature Cxcr4b reporter (red) can be detected in the leading ends of the regenerating tubule (green). Accumulation of Cxcr4b positive cells can also be detected distally from the regenerating region.

File Name: Supplementary Movie 7

Description: *Cxcr4b* up-regulation in leading cells. Only the RFP signal of the *cxcr4b:cxcr4b-TagRFP-sfGFP* reporter (red) is shown.

File Name: Supplementary Movie 8

Description: Knockdown of *myca* impairs the repair response. Time-lapse video-microscopy revealed that depletion of *myca* prevented the reversal of the collective cell migration (proximal side of the pronephros), resulting in a failure of the pronephric duct cells on the proximal side to resume contact to the posterior cells.

File Name: Supplementary Movie 9

Description: No repair after knockdown of *myca* within 8 hours after injury. Time-lapse video-microscopy revealed that depletion of *myca* prevented the reversal of the collective cell migration (proximal side of the pronephros), resulting in a failure of the pronephric duct cells on the proximal side to resume contact to the posterior cells.

File Name: Supplementary Movie 10

Description: Cell migration speed of the posterior lateral line primordium. Time-lapse video-microscopy was performed for 2 hours. The leading cell was tracked over a period of 2 hours, acquiring an image every 8 minutes.
